# Friend or Foe: Exploring the Relationship between the Gut Microbiota and the Pathogenesis and Treatment of Digestive Cancers

**DOI:** 10.3390/microorganisms12050955

**Published:** 2024-05-08

**Authors:** Monica Profir, Oana Alexandra Roşu, Sanda Maria Creţoiu, Bogdan Severus Gaspar

**Affiliations:** 1 Department of Oncology, Elias University Emergency Hospital, 011461 Bucharest, Romania; monica.profir@rez.umfcd.ro (M.P.); oana-alexandra.rosu@rez.umfcd.ro (O.A.R.); 2 Department of Morphological Sciences, Cell and Molecular Biology and Histology, “Carol Davila” University of Medicine and Pharmacy, 050474 Bucharest, Romania; 3 Surgery Clinic, Emergency Clinical Hospital of Bucharest, 014461 Bucharest, Romania; bogdan.gaspar@umfcd.ro; 4 Department of Surgery, Carol Davila University of Medicine and Pharmacy, 050474 Bucharest, Romania

**Keywords:** digestive system cancer, gut microbiota, dysbiosis, gut–organ axis, gastrointestinal tumor development

## Abstract

Digestive cancers are among the leading causes of cancer death in the world. However, the mechanisms of cancer development and progression are not fully understood. Accumulating evidence in recent years pointing to the bidirectional interactions between gut dysbiosis and the development of a specific type of gastrointestinal cancer is shedding light on the importance of this “unseen organ”—the microbiota. This review focuses on the local role of the gut microbiota imbalance in different digestive tract organs and annexes related to the carcinogenic mechanisms. Microbiota modulation, either by probiotic administration or by dietary changes, plays an important role in the future therapies of various digestive cancers.

## 1. Introduction

Gastrointestinal cancers represent a global health burden, accounting for 1 in 4 cancer cases and 1 in 3 cancer deaths worldwide. This group of malignancies includes cancer of the esophagus, stomach, liver, pancreas, colon, and rectum [1]. The clinical presentation of gastrointestinal cancers is highly variable, underlying the complexity of this pathology. Early detection through screening programs and advances in treatment strategies, including chemotherapy, radiotherapy, and immunotherapy, have improved the outcome of gastrointestinal (GI) malignancies. However, challenges in this field remain, especially in advanced stages, and high efforts are being put into better understanding the molecular mechanisms behind this pathology and exploring new treatment options.

The development and progression of gastrointestinal cancers are believed to be the result of intricate interactions between genetic and environmental factors. Several risk factors, such as smoking, alcohol consumption, high-fat diets, and obesity, have been identified through epidemiological studies [2]. In addition, several microorganisms, including bacteria and viruses, have been acknowledged for their role in tumor development, some being classified as class I carcinogens (e.g., *Helicobacter pylori*, Epstein–Barr virus, hepatitis B and C viruses, and Kaposi-sarcoma-associated herpesvirus) [3]. This relationship between cancer and microorganisms has led researchers to investigate the implications of the gut microbiome in carcinogenesis. Significant correlations have been made, including the relationship between *H. pylori* and gastric cancer (GC) and *Fusobacterium* species’ key role in colorectal cancer (CRC) cases. In addition, end products of gut microbial metabolism, like short-chain fatty acids (SCFAs), also play a key role in homeostasis and are implicated in GI cancer development and progression [4]. This article aims to review the effect the gut microbiome has on carcinogenesis and to discuss the possible implications it has on GI cancer treatment.

## 2. Role of the Gut Microbiota in Digestive Health

Emerging studies have shown a strong relationship between the gut microbiota and host health. The human gut microbiota contains millions of microorganisms, including bacteria, fungi, and viruses, all in perfect equilibrium with the host [5]. The dominant phyla that reside in the healthy gut microbiota are represented by Firmicutes, Bacteroidetes, Actinobacteria, Proteobacteria, Fusobacteria, and Verrucomicrobia, of which 90% is represented by the Firmicutes and Bacteroidetes phyla [6]. Dysbiosis represents any alteration in the gut microbial composition, which several studies have linked to gastrointestinal diseases, cardiovascular issues, metabolic disorders, and cancer [7].

The gut microbiota is primarily associated with the breakdown of dietary fiber, the fermentation and anaerobic degradation of proteins and peptides, the host’s production of glycoconjugates, the deconjugation and dihydroxylation of bile acids, the biosynthesis of vitamins B and K, isoprenoids, and cholesterol, the reduction of cholesterol, and the degradation of amino acids and xenobiotics to produce energy through metabolism, nervous system development, appetite control, and intestinal and immune system development [8].

The primary site of interaction between microbes and the host immune system is the gastrointestinal tract of humans [9]. The body’s defense mechanism against external threats is the immune system, which comprises various organs, tissues, and cells that cooperate intricately. It comprises several blood cells, including T, B, and dendritic cells (DCs); lymphoid organs, including bone marrow and lymph nodes; and compounds such as complement, cytokines, and antibodies [10]. The immune system helps maintain and restore homeostasis by eradicating pathogenic microbes and cancerous cells and aiding tissue repair following injury [11].

The gut microbiota is required for defense against infections, food decomposition, and fermentation. It does this by secreting antimicrobial peptides or competing with pathogens for nutrients and adhesion sites [12]. The most extensively studied gut microbiota metabolites in regulating the immune system are SCFAs. Acetic, butyric, and propionic acids are among the SCFAs produced when fiber ferments in the colon [13]. These SCFAs interact with host cells, penetrate the intestinal epithelium, and control immunological responses [14]. Apart from their metabolic roles, these gut metabolites perform several regulatory activities, such as inhibition in the production of the pro-inflammatory cytokine tumor necrosis factor (TNF) and the activation of nuclear factor-κB (NF-κB) [15]. Additionally, SCFAs block histone deacetylases (HDACs), which affect peripheral T cells, particularly regulatory T cells, and their function [16]. Certain SCFAs, like propionate and butyrate, also influence antigen presentation by preventing the growth of DCs via blocking HDACs [17,18]. Furthermore, butyrate and propionate are linked to preserving intestinal homeostasis through controlling the activity of hypoxia-inducible factor (HIF) [19]. This transcription factor, which is the main regulator of oxygen homeostasis when cells are affected due to hypoxemia, is the main link between maintaining the intestinal epithelial barrier, the stimulation of CD4+ T cells, and interleukin (IL)-22 production, and even the overexpression of mucins MUC2, MUC3 and intestinal trefoil factor (ITF), all essential in the epithelial restoration of the colon [20,21,22]. Therefore, by altering the HIF, which affects this crosstalk, SCFAs are crucial in controlling the host–microbe relationship.

Apart from SCFAs, other metabolites the gut microbiota generates, like indole derivatives originating from tryptophan and polyamines resulting from dietary arginine, play significant roles in immunomodulatory processes. By encouraging the growth of intestinal goblet cells and the release of mucins and antimicrobial peptides, indole derivatives support the integrity of the enteric epithelium and the body’s defense against microbes [13]. Additionally, tryptophan derivatives support the development and activity of regulatory T cells, innate lymphoid cells 3 (ILC3), and anti-inflammatory macrophages. Intestinal epithelial cells (IECs) are preserved by IL-22, which controls commensal microbiota equilibrium and guards against *Citrobacter rodentium* infection [23].

Due to the close connection between the human immune system and gut microbiota, abnormalities in either system might cause diseases. The equilibrium between identifying pathogens and preventing self-attacks is disrupted in autoimmune disorders [24]. As a result, the immune system continues to activate even without infection, and control over inflammation is lost [25]. Dysregulated immune responses, abnormally high levels of autoreactive T cells, autoantibody-producing B cells, and the increased production of pro-inflammatory cytokines are the hallmarks of autoimmune and inflammatory illnesses [26]. Thus, dysbiosis can lead to the appearance of some inflammatory and immunological disorders, including the well-known inflammatory bowel disease (IBD) [27].

Other functions of the gut microbiota that demonstrate its significance for preserving health include its ability to protect against infections [28]. Propionate and butyrate, in particular, are SCFAs that can suppress *Salmonella typhimurium*’s pathogenicity island 1 gene expression, which is necessary for intestinal epithelial cell invasion [29]. Furthermore, commensals can metabolize both host-derived and microbial-synthesized molecules. This can lead to the production of primary bile acids, which are then converted into secondary bile acids, which are essential for pathogen defense because they inhibit the growth of *Clostridium difficile*, for example [30].

The enteric neural system (ENS), which regulates major gastrointestinal functions apart from the central nervous system, can also receive additional focus. Recent research has demonstrated that butyrate can affect GI motility and brain functioning by expressing neuromodulator genes. Specifically, butyrate raises the amount of choline acetyltransferase via the Src-kinase signaling route, acetylates histone H3K9 in enteric neurons, and stimulates cholinergic pathways to improve colon motility [31].

Furthermore, in cancer, acetate and propionate cause necrosis at pH 5.5 and apoptosis at pH 7.5 in the human colon adenocarcinoma cell line (HT-29). These processes are most likely brought on by HT-29 cell’s accumulation of reactive oxygen species (ROS), inner membrane permeabilization, mitochondrial depolarization, and a sharp reduction in adenosine triphosphate (ATP) levels [32].

One of the main functions of the gut microbiota is mucosal and systemic immune regulation through CD4+ and CD8+ T-cell functions, inducing the needed reactions to defend the host from pathogens. When dysbiosis occurs, the immunological function is also affected, leading to chronic inflammation and carcinogenesis by activating inflammatory pathways [33].

## 3. Links between Dysbiosis and Digestive Cancers

GI cancers, the group of malignancies affecting the digestive system, represent a major global burden with high morbidity and mortality rates. Age, diet, viruses, different toxins, and genetic predisposition are among the risk factors associated with gastrointestinal diseases [34]. Efforts are being made to establish the mechanisms by which dysbiosis participates in GI cancer development and progression.

Carcinogenesis is a multi-step process where environmental risk factors and the host’s immunological status play a major role. The relationship between cancer and microorganisms, including viruses (e.g., Human Papillomavirus (HPV), Epstein–Barr virus, hepatitis B and C viruses, and Kaposi-sarcoma-associated herpesvirus) and bacteria (e.g., *H. pylori*) has been long established [35,36]. Current research suggests that microbial infections cause 20% of the cases of carcinogenesis, and microbial commensal imbalance is linked to various cancers [37].

Chronic inflammation, the production of microbial metabolites, and immune modulation are the main microbiota-related factors that research has linked to the development of GI cancers [38]. Inflammation is known to be the hallmark of cancer, causing deoxyribonucleic acid (DNA) damage and mutations that promote carcinogenesis. The association between inflammation and malignancies was first described by Virchow in the 19th century, underlying the fact that tumors frequently arose at the sites of chronic inflammation and that inflammatory cells were found in tumor samples [39]. ROS primarily causes inflammation-induced DNA damage via direct reaction or through reactive lipid peroxidation intermediates [40]. Numerous studies highlight the relationship between the microbiota and chronic inflammation and how pathogens induce inflammation and promote cancer development [28,33]. Bacterial infections can lead to aberrant DNA methylation via chronic inflammation, ultimately leading to carcinogenesis [41]. Pro-inflammatory cytokines such as TNF alpha, IL-6, and IL-1beta and transcription factors like NF-kappaB and STATs are increased in chronic inflammation and play a huge role in this process, leading to cancer development [42]. TNF alpha is secreted by inflammatory cells that infiltrate the tumor and activate oncogenic signaling pathways like NF-kappaB and the Wnt signaling pathway [42]. TNF alpha leads to chromosomal instability, gene mutations, and gene amplifications by producing reactive oxygen species (ROS) [43]. In addition, chronic inflammation can also inactivate tumor-suppressor genes like p53 and activate oncogenes such as KRAS mutation [44,45].

The sole presence of dysbiosis is not enough to cause cancer development. Dysbiosis-associated alterations, such as inflammation, immune response, and genotoxins produced by bacterial species in the gut and cellular stress, can potentially lead to carcinogenesis [46].

Major biological processes such as intercellular communication and signaling transduction are controlled, among other mechanisms, by the influence of bacterial metabolites. These mechanisms are important both in normal cells and cancer cells [47].

There are several bacterial metabolites that seem to play a role either in cancer cell suppression or progression (for a detailed review, see [48]). There is still a long way to go until science can generate comprehensive molecular and mechanistic insights. Some bacterial metabolites can exert direct and indirect genotoxic activity, such as hydrogen sulfide and p-cresol. These two metabolites can directly interact with the intestinal epithelial cells and with the stromal cells, playing a role in cancer progression

Indole, another metabolite, is shown to associate with AHR and promote inflammation after its nuclear translocation through the upregulation of IL-6 in colon cancer cells.

Some bacterial species directly inject their toxins into the host cells, inducing DNA damage. In this category, one can exemplify colibactin (produced by specific strains of E. coli) and cytolethal distending toxins (produced by *E. coli*, *Campylobacter jejuni*, *Helicobacter hepaticus*, and *Salmonella enterica*) [49]. In addition to DNA damage, genome stability is also influenced by the downregulation of genes involved in DNA repair, such as TP53, MSH2, MLH1, and ZRANB3 [50].

A mechanism involved in restricting tumor growth is altering the redox balance. This is the case for reuterin, which restricts tumor growth by inhibiting protein biogenesis and translation in ribosomes [51]. Some other microbiota-derived metabolites, such as taurine, histamine, and spermine, were shown to suppress inflammation in colon cancer [52].

The multi-step process of carcinogenesis is impacted by environmental risk factors and the host’s immunological status, with the gut microbiota and its metabolites playing a major role [53]. Certain pathogens can adversely affect the host’s metabolism, gut flora, and immune system, which can lead to cancer inside a dysbiotic gut. Interestingly, cancers can arise locally or remotely from dysbiotic conditions in the gastrointestinal system [54]. According to current research, 20% of the cases of carcinogenesis are caused by microbial infections, and microbial commensal imbalance is linked to a variety of cancers [37].

**Figure 1:**
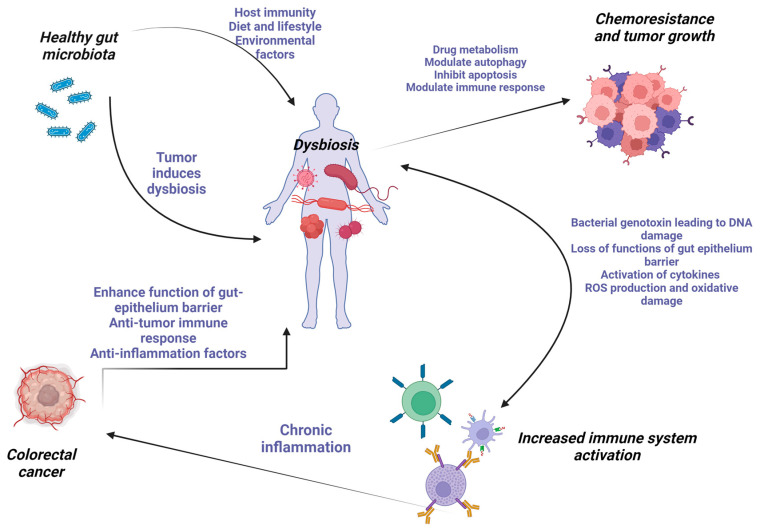
The interplay between dysbiosis and CRC development. Image created with Biorender.

Immune regulation is another major factor present in carcinogenesis. According to comparative studies conducted on germ-free mice, mice with dysbiotic microbiota have disturbed innate and adaptive immunological activities, resulting in an altered immune homeostasis [55,56]. The immune system recognizes pathogen-associated molecular patterns (PAMPs) like lipopolysaccharides and flagellin via Toll-like receptors (TLRs), which leads to cytokine production [57]. The adaptive immune system participates in carcinogenesis through T helper (Th) cells, T regulatory (Treg) cells, and B cells by secreting immunoglobulin A (IgA) [58]. Research has also demonstrated that gut-dwelling commensal and pathogenic bacteria directly influence systemic cancer immunity through immunoregulatory mechanisms [59]. In response, cancer cells release chemicals that alter the diversity and makeup of gut bacteria, controlling the tumor microenvironment (TME) and suppressing the immune system [60] (Figure 1).

As mentioned before, the gut microbiota plays a major role in intestinal metabolism, thus participating in energy production. The gut microbiota observed in patients with GI cancers refers to a shift in the population of microorganisms, with increased levels of carcinogenic-promoting bacteria (e.g., *H. pylori*, *Fusobacterium nucleatum*) and a decrease in the levels of health-promoting bacteria (e.g., *Roseburia* spp.). These differences in terms of the microbial population also affect the intestinal metabolome, a dynamic complex system that mirrors the interactions between the host, the gut microbiota, and the environment [61]. The gut metabolome refers to the small molecules and metabolites found in the intestinal lumen that are the end products of intestinal metabolism [62]. Studies have shown that certain metabolites, such as SCFAs, have protective roles and that lower levels have been associated with diseases, including GI cancers [63]. On the other hand, metabolites such as lipoteichoic acid and secondary bile acids promote carcinogenesis via excessive pro-inflammatory production and an increased cellular proliferation, respectively [64,65]. There is a strong relationship between the shift in gut microbial populations, metabolome alterations, immunological changes, and cancer. This connection has been demonstrated by Li et al. in a study that showed how gut microbiota influences cancer development and progression in a survey of Apc^min/+^ mice. Fecal microbiota transplantation (FMT) from CRC patients to Apc^min/+^ mice increased tumor proliferation and decreased apoptosis in tumor cells. This led to altering gut barrier function and an upregulated pro-inflammatory cytokine profile. FMT from CRC patients increased the levels of pathogenic bacteria and decreased the abundance of SCFA-producing bacteria and SCFA levels. In addition, it led to an increased expression of beta-catenin and cyclinD1, which indicated the activation of the Wnt signaling pathway [66].

Inflammation can be caused by bacterial components such as lipopolysaccharide (LPS), a component of the cell wall in Gram-negative bacteria, through interaction with pattern recognition receptors (PRRs), which can be found on the surface of immune cells [67].

Several studies brought evidence of the impact that gut microbiota alterations have on the immune system, and later on the development or digestive cancers or symptoms associated with CRC [68]. An increased number of CRC-associated chemokine genes helped slow down the CRC development by recruiting tumor-infiltrating lymphocytes (TILs) [69].

Interleukin-17 (IL-17) is a cytokine produced primarily by T helper 17 (Th17) cells and its role in cancer is complex and context-dependent, as it can have both pro-tumorigenic and anti-tumorigenic effects, depending on the specific cancer type, stage of disease, and the tumor microenvironment [70]. A relationship between IL-17 and breast, gastric, and prostate cancer has been established. However, the role of IL-17 in oncology is highly controversial and remains to be established [71,72,73,74,75]. In some contexts, IL-17 has been associated with promoting cancer progression by stimulating the production of pro-inflammatory cytokines and chemokines, such as IL-6, IL-8, and CXCL1, which contribute to inflammation and tumor growth [76]. IL-17 can also induce the expression of matrix metalloproteinases (MMPs) by tumor cells and stromal cells, facilitating tumor invasion and metastasis [77]. Furthermore, IL-17 can promote angiogenesis, forming new blood vessels that supply nutrients to tumors, thereby supporting tumor growth and progression [78].

Conversely, in other situations, IL-17 has been shown to have anti-tumorigenic effects like enhancing the recruitment and activation of immune cells, such as cytotoxic T cells and natural killer (NK) cells, which can recognize and eliminate tumor cells. IL-17 can also stimulate the production of anti-tumor cytokines, such as interferon-gamma (IFN-γ), which has potent anti-tumor activity [79,80]. Additionally, IL-17 has been implicated in promoting the formation of tertiary lymphoid structures (TLSs) within tumors, which can facilitate an effective anti-tumor immune response [81]. Studies show that blocking IL-17A improves the efficacy of anti-PD-1 immunotherapy in microsatellite-stable colorectal cancer mouse models [82]. Regarding its role in other cancers, research brings to light its important role in tumor proliferation in lung cancer [83]. However, there are also conflicting results from mice studies that revealed that in mice with lung cancer and dysbiosis, IL-17 was increasingly produced, which increased PD-1+T cell expression and the recruitment of neutrophils, which resulted in reduced survival and increased the burden of lung tumors [84]. There are recent evidence speaking of the gut–lung axis because specific bacterial strains were predominantly found in the gut of the patients suffering from non-small cell lung cancer (such as *Prevotella*, *Lactobacillus*, *Rikenellaceae*, *Streptococcus*, *Enterobacteriacea*, *Oscillospira*, and *Bacteroides plebeius*) [83] or small cell lung cancer (for example *Klebsiella*, *Acidovorax*, *Polarmonas*, and *Rhodoferax*) [85]. Understanding the interactions between the lungs and the gut and how they influence each other’s health and disease states could lead to novel therapeutic strategies for respiratory and gastrointestinal disorders. While there is robust scientific backing for directing interventions towards IL-17 as an immune target to mitigate the adverse effects of targeted therapy, with the potential to be curative and serve as a favorable prognostic indicator of an anti-tumor immune response, further experimental and clinical investigations are warranted to substantiate this strategy comprehensively and stop lung cancer dissemination [80].

## 4. Mechanisms of Microbial Influence

The altered metabolome in patients with gastrointestinal cancers is partially due to the differences observed in the tumor microenvironment. Generally, tumor cells are exposed to chronic hypoxia from an early stage in carcinogenesis, and extensive hypoxia has been associated with more aggressive tumors [86]. Cancer cells produce energy primarily through glycolysis rather than through the tricarboxylic acid (TCA) cycle, even with optimal oxygenation, which is an effect known as the Warburg effect [87]. In addition, the TME is characterized by hypoxic and hypermetabolic activity in glycolysis and glutaminolysis [88]. One microbiome and metabolome analysis on patients with gastric cancer reported differences in microbiome and metabolome profiles when comparing tumor tissues and non-tumor tissues, which may eventually impact gastric cancer development and progression [89]. The findings were consistent with the results of similar studies, reporting an enhanced relative abundance of carbohydrate conjugates and discriminative metabolites in the amino acid class in the tumor tissue compared to the non-tumor tissue, in addition to the increased levels of nucleosides [89,90,91]. The enhanced levels of amino acids, except for glutamine in gastric and colorectal cancer samples related to glutaminolysis, the autophagic protein degradation, and active glutamine degradation are used by tumor cells for energy production [92]. Glutamine is essential for cancer cell survival and controls the main regulator of protein translation mTORC1, which is required for the anabolic growth of cancer cells [93].

One study using chemical derivatization and chromatography/mass spectrometry identified 18 distinct metabolites between cancerous tissue and adjacent healthy tissue of gastric mucosa in patients with gastric cancer, such as higher levels of L-valine, L-isoleucine, serine, and propanoic acid in the malignant tissue compared to the non-malignant tissue. In addition, the authors identified five metabolites that are different between the invasive and non-invasive tumors, reporting higher levels of L-cysteine, hypoxanthine, and L-tyrosine and lower levels of phenanthrenol and butanoic acid [94].

Moreover, significant differences in serum metabolites were reported in patients with esophageal, gastric, and colorectal cancers compared to healthy controls. The serum levels of specific molecules that participate in the TCA cycle appeared to be upregulated in the patients with GI cancers, suggesting that TCA cycle disruptions might correlate with cancer cell proliferation, which calls for high energy levels. Variations in the levels of malonic acid and L-serine contributed to the separation of esophageal cancer. In contrast, gastric was primarily characterized by differences in the levels of 3-hydroxy propionic acid and pyruvic acid, and differences in the levels of L-alanine, glucuronic lactone, and L-glutamine enhanced the segregation of colorectal cancer. This study revealed that some of these metabolites possessed higher sensitivity and specificity in detecting early stages of malignancy compared to conventional biomarkers such as CA19-9 or CEA [95]. A recent metabolome analysis on patients with gastric cancer identified six microbial-related metabolites that could potentially be used for the diagnosis of gastric cancer: 6-methyl nicotinamide, aniline, L-kynurenine, lignoceric acid, methyl palmitate, and oleic acid [96].

Esophageal squamous cell carcinoma cases are frequently diagnosed in advanced disease stages due to the absence of specific symptoms and biomarkers. One study performed metabolomics analysis on both patients with esophageal cancer and healthy controls, aiming to identify biomarkers for early detection and prognosis. The authors reported abnormal amino acids and lipid metabolism, which are critical metabolic signatures of esophageal squamous cell carcinoma. Pathway analysis also highlighted that retinol and linoleic acid metabolism were significantly altered, and targeting these pathways may represent a new promising therapeutic approach [97].

Recent studies have also investigated the impact of SCFAs on gut health and their implications in GI cancers. SCFAs are fermentation end products produced by the gut microbiota, primarily represented by acetate, propionate, and butyrate. Their main purpose is to serve as a substrate for energy production after they have been absorbed by IECs via the monocarboxylate transporter 1 (MCT-1) and the sodium-coupled monocarboxylate transporter 1 (SMCT-1) [98].

In esophageal squamous cell carcinoma, the gut microbiota seems to be characterized by a decreased abundance of butyrate-producing bacteria and increased levels of carcinogenic and pro-inflammatory bacterial species [99]. Research has also demonstrated that butyrate and propionate can restore esophageal epithelial barrier lesions driven by IL-13 by increasing the expression of barrier proteins filaggrin (FLG) and DSG1 [100]. The decreased plasma levels of butyrate and propionate have also been reported in patients with gastric cancer [101].

A recent study has shown that butyrate inhibits the proliferation of gastric cancer cells and induces the apoptosis of gastric cancer cells via a mitochondrial pathway, thus demonstrating the anticarcinogenic effects of butyrate on gastric cancer cells [102]. These findings are also supported by Sun et al. in a study aimed to reveal the signaling network altered by butyrate in gastric cancer cells [103]. In addition, Clostridium butyricum has shown promising results when administered orally after a gastrectomy intervention by reducing early postoperative inflammation, increasing immune capacity, and reducing the occurrence of postoperative complications [104].

The increased levels of SCFAs have a protective role on intestinal health, and the decreased concentrations of butyrate, propionate, and acetate have been associated with an increased risk of developing colorectal cancer [105]. Butyrate induces anti-inflammatory effects by inhibiting histone deacetylases in intestinal epithelial cells and immune cells, thus downregulating pro-inflammatory cytokines IL-6 and IL-2 [106]. In addition, by downregulating the expression and functional activity of alpha2 beta1 integrin, butyrate can also induce apoptosis in CRC cells [107]. The anti-inflammatory effects of butyrate and propionate are also exerted through their capacity to regulate colonic T-cells, according to some animal studies [108,109].

Bacteriocins are a group of ribosome-synthesized cationic bacterial peptides produced by probiotics. Bacteriocins secreted by lactic acid bacteria have been initially studied for their food-preserving and antibacterial properties. However, recent studies have reported the anticarcinogenic capacities of bacteriocins. Nisin, enterocin, plantaricin, pediocin, bovicin, and microcins are among the most studied bacteriocins for their benefits in cancer treatment, nisin being the most investigated. Apart from their ability to inhibit cancer cells, bacteriocins also can differentiate cancer cells from normal cells [110]. For example, the bacteriocin LNS18 produced by an *Enterococcus* strain has demonstrated promising anticancer action against a model of liver cancer cells by inducing cellular ROS and arresting cells in the G0 phase without cytotoxic effects on normal cells [111]. Nisin, a bacteriocin produced by *Lactococcus lactis*, was shown to induce apoptosis in colorectal cancer cells by increasing the expression of pro-apoptotic protein BCL-2, shifting the BAX/BCL-2 apoptotic index [112]. One *Lactobacillus plantarum* strain was also reported to inhibit CRC cell growth while increasing the viability of healthy cells through the production of plantaricin, a bacteriocin-like compound [113]. These studies and other recent studies on the cytotoxic effects of bacteriocins on different cancer cell lines have been listed in Table 1.

However, despite having several advantages, bacteriocin mass production would be extremely expensive and few details about their pharmacokinetics and bioavailability are known [125,126]. Future research should focus on investigating bacteriocin intestinal distribution, half-life, and clearance. Cellular resistance mechanisms against bacteriocins represent another matter of concern. Some researchers worry that bacteriocin resistance could be passed to other cells similarly to the transfer of antibiotic resistance genes [127].

Diet has a strong impact on the composition of microbiotas and plays a key role in GI carcinogenesis. Animal studies have shown that a high-fat diet increases the abundance of *Clostridium* strains and decreases the levels of health-promoting bacteria such as *Escherichia*, *Shigella*, and *Lactobacillus* strains [128]. High-fat diets have also been shown to have a pro-inflammatory effect, leading to an increase in the levels of natural killer cells and T-cell recruiting factors [129]. Epidemiological studies have also shown that diets high in refined starches, eggs, cheese, and red meat are associated with an increased risk of CRC, whereas high fiber diet lowers the chances of developing CRC [130]. Increased fat intake promotes a procarcinogenic environment in mice via inducing leptin signaling and signal transducer and activator 3 (STAT3), favoring intracellular beta-catenin accumulation [131]. In addition, the relationship between obesity and an increased risk of malignancy is also acknowledged. By comparing rectosigmoid mucosal biopsies from premenopausal women before and after weight loss induced by a very low-calorie diet, a study has demonstrated that weight loss was associated with reduced inflammation and downregulated gene pathways. Thus, a reduction in TNF-alpha, IL-1beta, IL-8, and monocyte chemotactic protein concentrations and decreased T-cell and macrophage counts was reported. The downregulation of pro-inflammatory cytokine and chemokine pathways was also observed, along with the reduced expression of NF-kappaB and STAT3 transcription factors [132].

## 5. Digestive Cancers and the Microbiota

The human gut microbiota contains millions of microorganisms, including bacteria, viruses, and fungi, that exist in perfect equilibrium with the host [5]. Any disturbance in the gut microbiota composition resulting in an imbalance between protective bacteria and pathogens is regarded as dysbiosis, which is associated with increased disease occurrence [133]. Emerging studies have demonstrated that the microbiota in GI cancer tissue differs in terms of the distribution and microbial diversity, and metabolic function is predicted from the microbiota of adjacent non-malignant tissue. Generally, decreased microbial richness has been observed when analyzing malignant tissue from esophageal, gastric, and CR cancer; however, increased bacterial diversity has been reported in a cohort of hepatocellular carcinoma patients (HCC) [129,134,135,136,137,138].

Microbiome studies have helped identify the genera that are more consistently modified in GI cancers. In esophageal adenocarcinoma, strains of *Lactobacillus*, *Prevotella*, *Enterobacteriaceae*, and *Akkermansia muciniphila* are frequently enriched along with decreased levels of *Streptococcus pneumoniae* [129]. Studies assessing the animal and human gastric microbiota have reported *Helicobacter pylori* (*H. pylori*) dominated microbiota profiles in patients with gastritis and decreased levels of *Streptococcus*, *Prevotella*, and *Neisseria* species [135,139]. Interestingly, GC microbiota profiles are characterized by the loss of *H. pylori* species and bacteria that normally colonize the intestinal microbiota, such as Lactobacilli or Enterococci, and oral bacteria like *Fusobacterium* dominated the gastric microbiota of GC patients [135,140]. Alterations in the microbial composition also occur in CRC, with an increased abundance of pro-oncogenic bacteria such as *Fusobacterium nucleatum*, *B. fragilis*, *Enterococcus faecalis*, *E. coli*, *Porphyromonas*, *Peptostreptococcus anaerobic*, and *Micromonas parvum* and a decreased abundance of health-promoting taxa like Bifidobacterium and the butyrate-producing *Clostridium butycum* and *Roseburia* spp. [141].

*H. pylori* is a spiral-shaped Gram-negative bacterium that colonizes the gastric mucosa and has been classified as a class I carcinogen by the International Agency for Research on Cancer [142]. *H. pylori* infection has been linked to gastric ulcers, gastric cancer, mucosa-associated lymphoid tissue (MALT) lymphoma, and several extragastric conditions, including insulin resistance, type II diabetes, cardiovascular events, and iron deficiency anemia [143]. The capacity of *H. pylori* to induce GC is also enhanced by non-*H. pylori* bacteria, as demonstrated in one study on insulin–gastrin mice. Mice colonized with *H. pylori* and different species of intestinal bacteria had significantly higher GC rates than mice colonized with a specific pathogen-free flora with or without *H. pylori* colonization [144]. One of the mechanisms by which *H. pylori* induces inflammation and, thus, gastric cancer is the inhibition of gastric acid secretion via the inhibition of the cagPAI genes [145]. Hypochlorhydria can thus lead to the colonization of other inflammation-promoting pathogens, which was observed in patients with chronic atrophic gastritis [146,147]. Secondly, *H. pylori* infection leads to an exaggerated immune response, neutrophils and lymphocyte infiltration, and the production of pro-inflammatory cytokines such as IL-1, IL-6, IL-8, and TNF-alpha [147,148]. The production of IL-8 is also enhanced through the activation of the transcription factor NF-kappaB [148]. In addition, *H. pylori* induces the expression of the pro-inflammatory cyclooxygenase enzyme (COX-2), which promotes carcinogenesis via the inhibition of apoptosis, maintenance of cell proliferation, and stimulation of angiogenesis in cancer cells [149].

The Gram-negative bacterium *Fusobacterium nucleatum* is also an identified pro-inflammatory pathogen linked to human cancer [150]. *F. nucleatum* has been detected in the tumor samples of patients with colorectal cancer, and it has been shown that *F. nucleatum* can also activate NF-kappaB and induce tumor myeloid cell infiltration, which leads to a pro-inflammatory environment, linked to colorectal cancer in animal studies [125]. Interestingly, an increased abundance of *F. nucleatum* DNA has also been detected in esophageal cancer tissue and is associated with shorter survival rates and aggressive tumor behavior [126]. In 2013, Kostic et al. demonstrated that *Fusobacterium* is enriched in adenoma tissue compared to adjacent healthy tissue, suggesting the early role of the pathogen in tumorigenesis [125]. Recent studies have shown that the abundance of *F. nucleatum* increases from rectum to cecum and that high levels of *F. nucleatum* in tumor samples are correlated with poor survival rates in patients with colorectal cancer compared to patients with a low abundance of *F. nucleatum* [127,151]. Moreover, studies have demonstrated that a high abundance of *Fusobacterium* is associated with disease recurrence after chemotherapy treatment in CRC [152]. *Fusobacterium* has also been detected in liver metastases by in situ hybridization (ISH). Many liver metastases have a similar tumor microbiota to the microbiota of the primary lesion [153]. *Fusobacterium* also appears to induce chemoresistance to Oxaliplatin and 5-Fluorouracil (5-FU) via the autophagy pathway, decreasing the apoptosis effect of these highly used chemotherapy agents [152]. These findings support the concept that these patients might benefit from anti-fusobacterial therapy. However, one study reported an improved therapeutic response to anti-PD-L1 blockade by augmenting tumor sensitivity to anti-PD-L1 therapy, thus resulting in prolonged survival in mice treated with *F. nucleatum* [154].

ETFB and polyketide synthase positive (pks+) *E. coli* have also been associated with colorectal cancer development [155,156,157]. These bacteria induce DNA damage by producing colibactin and the *B. fragilis* toxin (BFT), respectively [158,159,160,161]. The levels of both ETFB and pks+ Escherichia coli have been reported to be significantly increased in the intestinal mucosa of patients suffering from familial adenomatous polyposis (FAP). Moreover, co-colonization can be found in more than half of the patients suffering from FAP, increasing tumor development [162]. According to in vivo studies by stimulating cell signal transduction pathways that lead to cell morphology changes and the cleavage of E-cadherin, BFT alters the intestinal barrier function and increases epithelial cell proliferation and cytokine expression, resulting in chronic inflammation via a Stat3 and TH17-dependent pathway [163,164]. In addition, colibactin leads to an altered anti-tumor T-cell response along with lower levels of CD3+ and CD8+ T cells and an increased inflammatory response in APC^min/+^ mice [165].

## 6. Microbiota as a Therapeutic Target

**Figure 2:**
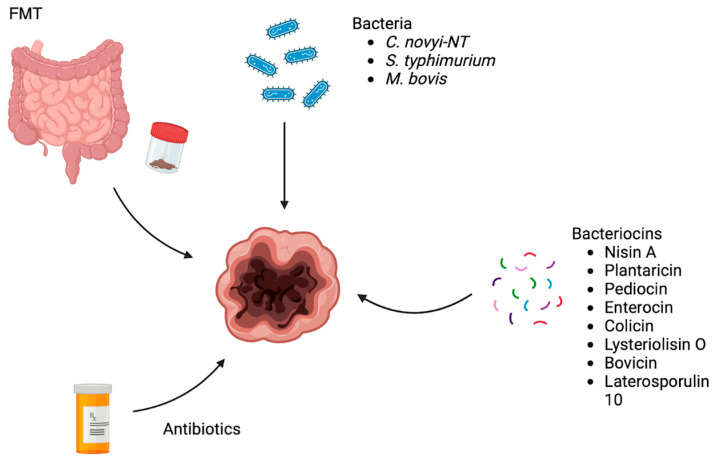
Gut microbiota modulation strategies for cancer therapy. Image created with Biorender.

Although great progress has been made, current treatment strategies for GI cancers, including surgery, chemotherapy, radiation therapy, and immunotherapy, are associated with high toxicities and adverse reactions. In addition, new anticancer regimens are continually needed due to the development of currently available chemotherapy agents. Seeing how disturbances in the gut microbiota composition can promote carcinogenesis and cancer progression, maintaining a healthy gut microbiota is essential for the prevention of malignancies. In recent years, researchers have been investigating ways to integrate the modulation of the gut microbiota in the treatment of several gut microbiota-related diseases including GI cancers. Current methods include probiotics, FMT, and microbiota-altering agents. These methods target the gut microbiota and their success rates are based on its capacity to modulate the immune response and cellular gene expression patterns [166]. The therapy methods discussed in this article are illustrated in Figure 2.

The gut microbiota plays a key role in cancer treatment, especially in the treatment with immune checkpoint inhibitors (ICIs), CTLA-4, and PD-1 inhibitors being the most frequently studied. Several studies have shown that the microbiota modulates anti-tumor immune responses through both innate and adaptive immunity [167,168]. In 2015, it was demonstrated that the success rate of anti-CTLA-4 therapy was dependent on distinct *Bacteroides* species and that germ-free mice did not respond to CTLA-4 blockade. In addition, through FMT with feces rich in *Bacteroides*, transplanted mice showed improved response to treatment with CTLA-4 inhibitor [169]. Three years later, one study reported significant differences in the gut microbial composition of melanoma patients undergoing anti-PD-1 and were considered responders compared to nonresponders. A higher alpha diversity and an increased abundance of bacteria from the *Ruminococcaceae* family along with the enrichment of anabolic pathways were reported in responders compared to nonresponders. These differences in microbiome composition led to an increase in CD4+ and CD8+ effector cells with preserved cytokine responses to anti-PD-1 [170].

The easiest way to modulate the gut microbiota is through diet changes, including the consumption of probiotics through food supplements or in the form of fermented foods like yogurt, kefir, kombucha, or sauerkraut [171]. The consumption of lactic acid bacteria has several health benefits including improving mucosal immune function and reducing intestinal inflammation [171,172].

Several studies have demonstrated the benefits of probiotic administration in the oncological setting. The administration of *Bifidobacterium longum* and *Lactobacillus johnsonii* to CRC patients prior to surgical intervention has led to a shift in gut bacterial composition and a higher expression of CD3, CD4, CD8, and naive and memory lymphocytes compared to the placebo group [173]. Similarly, the administration of a probiotic mixture containing six strains of *Lactobacillus* and *Bifidobacterium* in CRC patients four weeks after surgery led to a reduction in pro-inflammatory cytokines TNF-alpha, IL-6, IL-10, IL-12, IL-17A, IL-17C, and IL-22 [174]. The benefit of probiotic administration in cancer patients has also been demonstrated for HCC patients. Apart from its capacity to shift the microbiota composition, the subcutaneous tumor inoculation of the probiotic mixture Prohep was effective in reducing HCC growth in animals. The authors reported reduced Th17 and IL-17 and decreased angiogenesis suppressing tumor growth in Prohep-treated mice [175].

Moreover, probiotic administration could also reduce the GI secondary effects of chemotherapy or radiotherapy, like nausea, vomiting, diarrhea, or constipation. A randomized controlled trial (RCT) on a group of oncological patients undergoing pelvic radiation therapy reported a reduction in diarrhea grade when a probiotic mixture containing *L. acidophilus* and *B. longum* was administered before radiotherapy [176]. Probiotic administration is generally considered safe. However, caution is advised in immunocompromised patients as it could lead to sepsis development [177]. Future studies about optimal dosage for specific probiotic strains and studies that evaluate the safety of these probiotics in cancer patients are needed.

FMT has been receiving increased attention in the past years. Several studies have proven its efficacy in digestive diseases like IBDs, irritable bowel syndrome, and some neurological conditions [178,179,180]. FMT has also been explored to improve the success rate of immunotherapy in patients with cancer [181,182]. Potential applications for FMT in oncology include the management of acute toxicities and the management of secondary complications [183]. However, there is a certain reluctance to choose FMT due to the possible adverse reactions and its high risk for infection, which is especially higher in immunocompromised patients.

FMT was shown to reverse dysbiosis in mice following antibiotherapy and chemotherapy with 5-FU, marking a significant increase in species with anti-inflammatory properties [184]. The benefit of FMT in cancer patients might be linked to its capacity to restore the balance of Toll-like receptor (TLR) signaling pathways. In addition, BALB/c mice implanted with syngeneic CT26 CR adenocarcinoma cells received FOLFOX (5-FU, leucovorin, and oxaliplatin) and FMT before and after the chemotherapy course. While the FOLFOX regimen induced diarrhea and intestinal injury, FMT efficiently reduced these symptoms and restored the gut microbiota composition. The mechanism might involve the gut microbiota TLR-MyD88-MF-kappaB signaling pathway [185].

The application of FMT in patients receiving immunotherapy is probably the most promising. A study conducted in 2019 investigated anti-PD-1 efficacy in two metastatic melanoma patients who failed at least one anti-PD-1 line of treatment after receiving FMT from melanoma patients with durable complete response to Nivolumab. The authors reported the overall safety of the procedure and the increased infiltration of antigen-presenting CD8+ T-cell in the tumor [186]. In the results from a recently published phase I clinical trial investigating the potential of FMT to overcome resistance to ICIs in patients with refractory melanoma, FMT from healthy donors increased anti-PD-1 efficacy, and the objective response rate was 65%, with four complete responses. The primary endpoint of this phase I clinical trial was met, with no grade 3 adverse events reported [187].

FMT is generally considered safe, with typically mild and transient adverse events such as diarrhea, abdominal cramps, low-grade fever, bloating, flatulence, and constipation [188]. However, serious adverse events like infections and GI complications have been reported [189]. Concerns about FMT in immunocompromised patients have been raised due to the perceived risk of translocation and sepsis [183]. However, Hefazi et al. demonstrated that FMT is effective and safe in oncological patients with recurrent C. difficile infection [190]. In addition, the results of one systematic review show that FMT has comparable safety in immunocompromised patients and immunocompetent individuals [191]. To eliminate infection risk, rigorous screening of donors and fecal material is essential [192]. While FMT has shown efficacy in certain conditions, its clinical applications are still evolving, and further research is needed to determine its effectiveness in various diseases. The challenges and limitations of FMT include the variability of donor material, the absence of standardized protocols, and the potential long-term effects on recipient health [193]. Future robust clinical trials are needed to establish the efficacy of FMT in different patient populations. In addition, ethical considerations surrounding donor selection, informed consent, and the long-term monitoring of recipients should be addressed [194]. Ensuring safety, standardizing protocols, and further research are essential for optimizing the use of FMT and expanding its potential applications in clinical practice.

The administration of antibiotics to kill or suppress pathogenic microorganisms has also been explored. For example, some researchers support that anti-fusobacterial therapy could benefit malignancies with a high *Fusobacterium* load. One in vitro study demonstrated that metronidazole reduces the *Fusobacterium* load and decreases cancer cell proliferation and tumor growth [153]. However, further studies are needed considering that metronidazole is a broad-spectrum antibiotic able to target several health-promoting gut bacterial species.

The role of specific bacteria in mitigating cancer has also been investigated. Although several bacterial strains have been associated with cancer development, bacteria have also demonstrated great potential for anticancer therapy [195]. Strains of *Clostridium*, *Bifidobacteria*, *Salmonella*, *Streptococcus*, *Mycobacterium*, *Lactobacillus*, *Escherichia*, *Caulobacter*, *Listeria,* and *Proteus* have the potential to target cancer cells, which derives from their capacity of colonizing the hypoxic tumor microenvironment and suppress growth in cancer cells [195,196] Obligate anaerobic bacteria like *Clostridium* spp. increase only in anoxic regions. Thus, they will only proliferate in the hypoxic areas of tumors when delivered as spores [197]. Therefore, small tumors or metastases might benefit more from the administration of facultative anaerobes, such as *Salmonella* and *Escherichia*, because they are better oxygenated [198].

*Clostridium novyi*-NT is an obligate anaerobe with promising anti-tumoral properties. The non-toxic form is obtained by eliminating toxin A, the primary toxin of *Clostridium* bacteria [199]. The anticancer properties of *C. novyi*-NT were first reported as early as 1935 when the study’s authors demonstrated that an enzyme produced by C. novyi-NT diminished tumor growth [200]. Preclinical animal studies have underlined the promising features of *C. novyi*-NT as an anticancer therapy [199]. More recently, studies have shown C. novyi-NT to have anti-tumoral activity in glioblastoma animal models [201]. In addition, C. novyi-NT is the only bacteria tested in humans for its antineoplastic effect [202]. Intratumoral administration in one patient with advanced leiomyosarcoma resulted in tumor shrinkage in the bone and the surrounding tissue [202]. C. novyi-NT induces tumor regression through the colonization of the hypoxic tumor microenvironment followed by the secretion of enzymes such as lipases and proteases, direct competition for nutrients and triggering host inflammatory responses [196].

*Salmonella enterica Serovar Typhimurium* is an enteric pathogen observed in both humans and animals, which causes typhoid fever [203]. *S. typhimurium* is able to grow in both aerobic and anaerobic environments, which makes it capable of targeting hypoxic tumors as well as non-hypoxic tumors and metastasis through the circulatory system [204]. The antineoplastic effect of attenuated *S. typhimurium* has been reported in several animal studies [205,206,207]. The inoculation of the attenuated *Salmonella* strains in a mice melanoma model led to tumor growth suppression and significantly increased survival rates compared to untreated mice [206]. In addition, VCMO1, a vaccine based on live attenuated *S. typhi* carrying a plasmid encoding expression of VEGFR2 was shown to inhibit the angiogenesis process in patients with locally advanced or metastatic pancreatic cancer [208]. The vaccine has successfully passed the phase I clinical trials [209]. The mechanism by which the bacterium inhibits tumor growth is not well defined; however, the recruitment of innate and adaptive immune system cells is the main mechanism according to some authors [210,211]. *Salmonella* administration increases the activation of CD8+ T cells and the number of activated NK cells, which leads to tumor regression [210]. In addition, Lee et al. demonstrated that S. typhimurium activates the autophagic signaling pathway via the downregulation of the AKT/mTOR pathway [212]

Another example of bacteria with anti-tumor effects is *Mycobacterium bovis* BCG, a strain used as a tuberculosis vaccine since 1921. This was demonstrated by Morales et al. in 1976 on a group of nine patients with recurrent superficial bladder tumors [213], followed in 1990 by the FDA approval of BCG as a treatment strategy for bladder cancer [214]. BCG exerts its anticancer effects through a combination of mechanisms, such as direct interaction with cancer cells, the activation of innate immune cells, and the activation and recruitment of tumor-specific CD4+ and CD8+ T cells [215].

Recently, one study demonstrated the antiproliferative activity of *Lactococcus lactis* strain Lc4 on CRC cell lines via the release of arginine deiminase (ADI), a cytostatic agent [216].

Despite considerable progress in the field of bacteria-based anticancer therapy, there are still many unanswered questions regarding the use of bacteria for antineoplastic purposes [217]. For example, the intrinsic bacterial immunogenicity and toxicity represent a safety risk for oncological patients due to the proliferation capacity of live bacteria and the potential to lead to systemic inflammation and septic shock. A second concern is the clearance of bacteria after the tumor has been cured. Although antibiotic therapy sounds like the logical answer, the negative impact of excessive use of antibiotics should also be considered. Thus, future research should focus on bacterial bioengineering to improve therapeutic efficacy and decrease the risk of adverse events. There are a number of ongoing Phase 1 studies investigating the safety of bacteria in cancer treatment, and the outcomes of these clinical trials ought to pave the way to new treatment strategies for oncological patients [218,219,220,221].

## 7. Challenges and Future Directions

Personalized cancer treatment strategies may involve considering the gut microbiota composition of individual patients. Understanding how specific microbial communities influence cancer progression and response to therapy could inform the development of tailored interventions.

In conclusion, the relationship between gut microbiota and digestive cancers is complex and multifaceted, with implications for both cancer pathogenesis and treatment. Continued research in this area promises to uncover novel insights and therapeutic avenues for combating these deadly diseases.

## Figures and Tables

**Table 1:** Studies of bacteriocins targeting different solid cancer cell lines.

Bacteriocin	Bacteria of Origin	Cell Line	Type of Cancer	Effect
Nisin A	*Lactococcus lactis*	HNSCC	Head and neck squamous cell carcinoma	Preferential apoptosis and decreased cell proliferation via the activation of CHAC1 [114].
		SW480	CRC	Increased apoptotic index (bax-bcl-2 ratio) and decreased proliferative impact [112].
		LS180, SW48, HT29, and Caco2	CRC	Reduces cell proliferation The downregulation of metastatic genes [115].
		HuH-7 and SNU182	Hepatocarcinoma	Decreased cell proliferation and activated cell apoptosis. Interfered with the mechanisms of drug resistance [116].
Plantaricin	*Lactobacillus plantarum*	E705 data	CRC	Decreased the proliferation of CRC cells [113].
		SW480, Caco-2, and HCT-116	CRC	Cancer cell death via a caspase-dependent pathway [117].
Pediocin	*Pediococcus acidilactici*	HepG2 and MCF7	Hepatocarcinoma Cervical adenocarcinoma	The inhibition of cell proliferation The induction of programmed cell death [118].
		HT29 colon adenocarcinoma and HeLa cervical cancer cells	Colon adenocarcinoma Cervical cancer	The inhibition of cell growth [119].
Microcin	*Klebsiella pneumoniae*	HT29 and SW620 cell lines	CR Adenocarcinoma	Decreased cell viability Significant tumor size reduction [120].
Enterocin colicin	*Enterococcus* strains *E. coli colicinogenic* strains	AGS	Gastric cancer	Increased the expression of apoptosis genes [121]
Listeriolysin O	*Listeria* *monocytogenes*	MCF-7 and SKBR-3	Breast cancer	The combination of LLO with the B3 antibody forms an immunotoxin with a potent cytotoxic effect [122]
Bovicin	*Streptococcus bovis*	MCF-7 and HEPG2	Breast cancer HCC	Cytotoxic effect [123]
Laterosporulin 10	*Brevibacillus laterosporus* SKDU10	MCF-7, HeLa, HT1080, and H1299	Breast cancer Cervical cancer Fibrosarcoma Lung carcinoma	Apoptotic and necrotic cell death [124]
Reuterin	*Lactobacillus reuteri*	HCT116, SW480, RKO, DLD1, B16, YUMM1.7, MIAPACA, and PaTu8988t	CRC Melanoma PDAC	Cell death via induced oxidative stress and Glutathione depletion [51]

AGS—gastric cancer cell line; B16, YUMM1.7—melanoma cell line; CRC—colorectal cancer; CR—colorectal; HNSCC—Head and neck squamous cell carcinoma; HCC—hepatocellular carcinoma; HuH-7, SNU182, HepG2—Hepatocarcinoma cell lines; HT1080—fibrosarcoma cell line; H1299—lung carcinoma cell line; LLO—Listeriolysin O; MCF7, HeLa—cervical cancer cell lines; MCF-7, SKBR-3—breast cancer cell lines; MIA PaCa, PaTu8988t—pancreatic adenocarcinoma cell lines; PDAC—pancreatic ductal adenocarcinoma; SW480, LS180, HT29, Caco2, HCT-116, SW620, RKO, DLD-1—colorectal cancer cell lines.

## Data Availability

Not applicable.

## Abbreviations

ATP	adenosine triphosphate
ADI	arginine deiminase
BFT	*B. fragilis* toxin
COX-2	cyclooxygenase enzyme
CRC	colorectal cancer
DCs	dendritic cells
DNA	deoxyribonucleic acid
ENS	enteric neural system
ETBF	enterotoxigenic bacteroides fragilis
FAP	familial adenomatous polyposis
FLG	filaggrin
FMT	fecal microbiota transplantation
5-FU	5-Fluorouracil
GC	gastric cancer
GI	gastrointestinal
HCC	hepatocellular carcinoma patients
HDACs	histone deacetylases
HIF	hypoxia-inducible factor
*H. pylori*	*Helicobacter pylori*
HPV	Human Papillomavirus
IBD	inflammatory bowel disease
ICIs	immune checkpoint inhibitors
IECs	intestinal epithelial cells
IL	interleukin
ILC3	innate lymphoid cells 3
IFN-γ	interferon-gamma
ISH	in situ hybridization
ITF	intestinal trefoil factor
IgA	immunoglobulin A
LPS	lipopolysaccharide
MALT	mucosa-associated lymphoid tissue
MCT-1	monocarboxylate transporter 1
MMPs	matrix metalloproteinases
NF-κB	nuclear factor-κB
NK	natural killer
PAMPs	pathogen-associated molecular patterns
pks+	polyketide synthase positive
PRRs	pattern recognition receptors
RCT	randomized controlled trial
ROS	reactive oxygen species
SCFAs	short-chain fatty acids
SMCT-1	sodium-coupled monocarboxylate transporter 1
STAT3	signal transducer and activator 3
TCA	tricarboxylic acid
TILs	tumor-infiltrating lymphocytes
TLR	Toll-like receptor
TLSs	tertiary lymphoid structures
TME	tumor microenvironment
TNF	tumor necrosis factor
